# Population Coverage to Reach Universal Health Coverage in Selected Nations: A Synthesis of Global Strategies

**Published:** 2019-06

**Authors:** Minoo ALIPOURI SAKHA, Najmeh BAHMANZIARI, Amirhossein TAKIAN

**Affiliations:** 1. Department of Global Health and Public Policy, School of Public Health, Tehran University of Medical Sciences, Tehran, Iran; 2. Department of Healthcare Management & Economics, School of Public Health, Tehran University of Medical Sciences, Tehran, Iran; 3. Health Equity Research Center, Tehran University of Medical Sciences, Tehran, Iran

**Keywords:** Population coverage, Universal health coverage, Global strategies

## Abstract

**Background:**

This study aimed to provide tailored transferrable lessons for expanding population coverage through a descriptive lens by reviewing the population coverage policies, reforms and strategies in selected nations.

**Methods:**

In this comparative short communication, 14 countries with different status of population coverage and political economy that had successful experiences with coverage expansion were selected and categorized in four groups to study their approaches to reach Universal Health Coverage (UHC).

**Results:**

Although each country needs to tailor its policies and reforms based on its own contextual factors, the legal right of citizens to social security and health protection are enshrined in most countries’ Constitution. Some countries adapted political and economic reforms to evolve their Social Health Insurance schemes. National laws to push governments to adapt UHC as a national strategy for ensuring that every resident is enrolled in health insurance schemes are key policies to reach UHC.

**Conclusion:**

A series of reforms are required to provide total population coverage through various approaches. To create an effective insurance coverage, physical merger of all insurance funds is not necessarily required. Further, the share of GDP for health is not a definite indicator to reach UHC. Finally, strong political commitment and citizens’ participation are the key issues in reaching UHC, while considering the poorest, remote and neglected population really matters.

## Introduction

To achieve the 2030 agenda for Sustainable Development, countries need a comprehensive strategy to invest in health, education, nutrition, environment, and security for all. These goals cannot be met without ensuring universal access to affordable and high-quality health services. Goal three of the sustainable development indicates achieving health and well-being at all ages. Universal Health Coverage (UHC) is a key target of SDGs, urging all countries to be well-positioned by 2030 to ensure universal health coverage for all citizens (1).

WHO recommends UHC aiming to provide universal and equitable access to health services for ensuring improved health outcomes. UHC consists of three interrelated components: (a) the full spectrum of health services according to need; (b) financial protection (2) from direct payment for health services when consumed; and (c) coverage for the entire population (2).

Population health coverage is defined as access to needed health services of good quality (3). Providing effective population coverage in UHC requires several steps including identifying poor and unprotected groups, empowering and informing them regarding their benefits, and ensuring their physical and financial access to required healthcare services (4).

Although universality suggests that all people are ideally covered, the World Health Report of 2010 noted that none of the high-income countries— that are commonly said to have achieved universal coverage—actually cover 100% of the population for 100% of the services available and for 100% of the cost and with no waiting lists (5). UHC is not a far-reaching goal for developing countries. Countries like Turkey, Mexico, Thailand, and Chile have had great ambition of UHC and their leaders developed national plans and strategies to prioritize and implement UHC (6). The overall aim of this study was to provide tailored transferrable lessons for expanding population coverage towards UHC in various settings.

## Methods

In this descriptive-comparative study, we reviewed the strategies and policies within 14 selected countries that had successful experiences with coverage expansion and studied their current population coverage policies and strategies. Included countries were categorized into four groups based on their various status of population coverage, political economy, economic situation and political commitment towards UHC (Table 1).

**Table 1:** Characteristics of selected countries

***Variable***	***Group 1***	***Group 2***	***Group 3***	***Group 4***
Status of population Coverage	Not universal	Coverage for a significant share of the population	Universal population coverage	Universal population coverage
Status of Political Economy	Strong commitment to UHC, middle and upper income (except Vietnam), emerging economies, strong programs in progress	Strong commitment to UHC, 40% of the world’s population and economy, sub-stantial health-system reforms, common health challenges	Strong political commitment to UHC despite massive political changes, strong political leadership, new investments, UHC policy reforms	Advanced political & economic power, OECD countries, mature systems and programs
Countries	Vietnam, Mexico, Chile	BRICS	Turkey, Thailand	Japan, England, Australia, South Korea

We searched the official websites of WHO and World Bank. We sought for publicly available reports and documents from the year 2000 until Nov 2016 and used general terms representing “population coverage” and “universal health coverage”.

In addition, we searched the Internet search engine Google to find additional documents and reports and enhance the comprehensiveness of our search. The reference lists of all selected documents were also scanned. Finally, among 47 reports and documents, 25 were selected and reviewed to extract related information about their policies, strategies and rules to expand population coverage to reach UHC. We describe countries’ approaches to reach universal population coverage in the following section and then discuss the transferrable lessons.

## Results

The legal right of citizens to social security and health insurance are preserved in most countries’ Constitution. Some countries adopted political and economic reforms to evolve their Social Health Insurance schemes. Increasing the proportion of the population enrolled in health insurance is a key policy goal for the governments. Other countries adopted UHC as a national strategy to ensure that every resident gets access to affordable health care (7–15). The main strategies, plans and policies are described in Table 2.

**Table 2:** Selected healthcare system indicators and population’ coverage, strategies and policies for 14 countries

***Groups***	***Countries***	***Population (thousands)***	***%THE/GDP***	***%OOP/THE***	***HDI (Human Development Index)***	***Strategies and policies***
**1**	Vietnam	93 448	7.1	36.8	0.683	Country’s constitution assures the right of citizens to health protection. After the political and economic reforms, national health insurance (contributory program) is launched for the formally employed, pensioners, and civil servants in 1993. The poor, ethnic minorities and the disadvantaged were covered by a Health Care Fund in 2003 (noncontributory). HCFP was merged into the national insurance (13, 14).
	Mexico	127 017	6.3	44	0.762	Health insurance comprises three subsystems: Social Security for salaried workers in the formal sector; the Social Protection System in Health (SPSS) offers health insurance to those not covered by any of the social security schemes; and the private system (12).
	Chile	17 948	7.8	31.5	0.847	The National Health Fund: 4 groups of the formally employed and the indigent (A through D), and combined all beneficiaries in the same risk pool after the 1981 reform. Since 1981 (ISAPERS), private insurers, have been participated in social health insurance scheme. Since 2005, the AUGE reform has been provided an explicit benefits package for all Fonasa and IsApril es beneficiaries (11).
**2**	Brazil	207 848	8.3	25.5	0.754	After health reforms in Brazil, health was enclosed a constitutional right in the late 1980s. Independent financing and service provision systems were unified and integrated into a single publicly funded system covering the whole population. Private health insurance covers around 25% of the population (7, 10, 16, 17).
	The Russian Federation	143 457	7.1	45.9	0.804	There is mandatory health insurance covering outpatient and inpatient care, except for tertiary and specialized healthcare. Military personnel and prisoners are excluded. Private voluntary schemes cover around 10% of the population (7, 16)
	India	1 311 051	4.7	62.4	0.624	Population coverage comprises of three subsystems: The National Rural Health Mission (NRHM): a state government-run primary healthcare system launched in 2005 states (18); Rashtriya Swasthya Bima Yojana (RSBY): provides health insurance coverage for inpatient treatment, and Yojana Rajiv Aarogyasri: focuses primarily on tertiary coverage. Private insurance seems to expand in the coming years (7, 16).
	China	1 376 049	5.6	32	0.738	In 1998 the Urban Employee Basic Medical Insurance focused on formal sector workers. In 2003, the New Cooperative Medical Scheme, offering subsidized health insurance for China’s rural population was introduced. In 2007, The Urban Resident Basic Medical Insurance for informal sector workers, children and the elderly in urban areas was launched. In 2009, commitment to providing affordable and equitable health care for all by 2020 is assured. A Medical Financial Assistance System was established to cover elderly patients, severely disabled people and seriously ill patients in low-income families (16, 19). In 2013, China is encouraging development of private health insurance (20).
	South Africa	54 490	8.8	6.5	0.666	In 2005, a pro-poor health insurance scheme was implemented for government employees (21). In 2015, to fulfill the constitutional obligation of the right to health, White paper on the implementation of National Health Insurance (NHI) to achieve UHC was published (7, 16). According to the paper, NHI will extend coverage to all South Africans with the priority of the poor and vulnerable groups. Private insurance covers 17% of the population (22).
**3**	Turkey	78 666	5.4	17.8	0.767	Health Transformation Plan has launched since 2003 in order to increase access to adequate health care for all. Turkey eliminated fragmentation in financing by merging the health insurance schemes into a Universal Health Insurance scheme. Primary health care services are provided free of charge. Under the mandatory UHI program, individuals are classified into one of four income groups with varying insurance premium. Turkey’s Integrated Social Aid Services System,” managed by the Ministry of Family Affairs and Social Policies helps the government to identify the poor (9).
	Thailand	67 959	4.1	11.9	0.740	Thailand achieved Universal Health Coverage through three major health insurance schemes: The Civil Servant Medical Benefit Scheme for civil servants and their dependents (8%), Social Health Insurance for formal sector and under the Social Security Act (16%), and the Universal Coverage Scheme for those not enrolled in CSMBS and SHI (76%). without merging three insurance schemes, these public purchasers - separated through a purchaser-provider split - manage financing system (8, 23).
**4**	Japan	126 573	10.2	13.9	0.903	Health insurance system consists of two complementary structure employment-based health insurance and residence-based health insurance. Employees of large companies and employees of small to medium companies are covered by the employment-based scheme, which receives no subsidy and 16.4% subsidies respectfully. Residence-based mandatory health insurance programs cover the self-employed, non-employed and pensioners below 75, which gets 50% subsidies. There is a health insurance program for elderly. People at the age of 40 and over are mandated to enroll in long-term care insurance (1).
	England	64 716	9.1	9.7	0.909	The NHS provides universal access to healthcare to all residents, the entitlement is based on clinical need, not on ability to pay. The Consumer demand for private health insurance is growing rapid (24). In 2015, about 11% of the England population had private voluntary health insurance that is less than many OECD countries. Also, over time, some user charges have been introduced (25, 26).
	Australia	23 969	9.4	18.8	0.939	Medicare, a universal public health insurance program administered by the federal government was instituted in 1984. This scheme was introduced to provide free treatment. Government policies encourage enrollment in private health insurance, which offers wide range of health services, more choice of providers, and quick access for nonemergency services (27).
	South Korea	50 293	7.4	36.1	0.901	The Population coverage achievement started modestly in 1977. First employees of large corporations with more than 500 workers were covered and then this coverage was extended to smaller firms. Medical Aid Program began for the poor, school teachers and government employees. Universal health coverage was achieved by expansion of health insurance to the rural self-employed and the urban self-employed in 1989. The first priority has been given to population coverage rather than the scope and depth of service coverage (28).

## Discussion

This study identified various pathways taken by countries belonging to diverse sociopolitical economies to reach universal population coverage, resulted in various UHC stages. Moving toward universal coverage is a procedure involves a range of challenges and points to consider. The importance of population coverage to achieve UHC is clear. Population coverage is a gradual, incremental and contextual-based process that cannot be achieved overnight. To move forward, a sequence of other reforms is essential. The experiences of Turkey and BRICS countries show that moving towards UHC is not fast and requires some fundamental problems in the health systems to be resolved.

There is no single and magic formula that fits all settings to expand coverage. Hence, countries should adapt policies and measures according to their contextual factors. As a fundamental issue under discussion in many countries, physical merger of various insurance funds are not necessarily required to reach UHC. For instance, Thailand reached UHC without physical merger of the existing three main funds.

To reach UHC, a clear statement of objectives and focus on visible outcomes is essential, followed by monitoring and evaluation of actions to complete the procedure. Foremost, total expenditure on health as a percentage of GDP is not necessarily a good indicator of reaching UUC. Thailand is an excellent example here. Thailand spent about 4.1% of its GDP on health (29), which is considerably lower than many other middle and high-income countries that spend around 7%–11% of its GDP on health. Besides, strong political commitment and support of influential stakeholders, as well as citizens’ participation, are the key issues to take sustainable policies and plans towards UHC. Brazil emphasized on health as a right of citizens and showed high level of engagement by the civil society and high commitment of relevant authorities to adapt related health policies to change the pathway, development, and implementation of its population coverage’s programs.

## Conclusion

Many countries have accepted UHC as a part of 2030 agenda and have begun to expand population coverage to fulfill this goal. The poorest populations who live in rural and remote areas often face the highest financial burden and health risks and are in a greater need for more services. Contextual-based and tailored interventions and policies are essential to expanding population coverage towards paving the way to UHC. Looking at the experience of various countries summarized in this article may provide practical transferrable lessons for many countries around the globe that are planning to expand population coverage to reach UHC by 2030.

## Ethical considerations

Ethical issues (Including plagiarism, informed consent, misconduct, data fabrication and/or falsification, double publication and/or submission, redundancy, etc.) have been completely observed by the authors.
